# IgG4-Related Disease (IgG4-RD) with Unique Combined Generalized Skin Rashes and Biliary Tract Manifestation: A Comprehensive Immunological Analysis

**DOI:** 10.3390/dermatopathology11030023

**Published:** 2024-07-16

**Authors:** Ye La Jung, Sudhanshu Agrawal, Beverly Wang, Sudhir Gupta

**Affiliations:** 1 Program in Primary Immunodeficiencies, Division of Basic and Clinical Immunology, University of California at Irvine, Irvine, CA 92697, USA; yela_jung@hotmail.com (Y.L.J.); sagrawal@hs.uci.edu (S.A.); 2 Department of Pathology and Laboratory Medicine, University of California, Irvine, CA 92868, USA; bevwang@hs.uci.edu

**Keywords:** IgG4-RD, regulatory B cells, CD4 Treg, CD8 Treg, T follicular regulatory cells, follicular helper T cells

## Abstract

IgG4-RD is a multisystem fibroinflammatory disease characterized by the infiltration of tissues by IgG4 plasma cells. Combined skin and biliary tract involvement in IgG4-RD has not been described. We present perhaps the most comprehensive analysis of lymphocyte subsets in the first case of IgG4-related generalized skin rash and first case of combined skin and biliary tract manifestations. A 55-year-old male presented with painful jaundice and generalized macular pigmented pruritic eruptions, and CT abdomen revealed biliary obstruction. Ampulla and skin biopsies were subjected to histology and immunostaining. Naïve, central memory (T_CM_), effector memory (T_EM_), terminally differentiated effector memory (T_EMRA_) subsets of CD4+ and CD8+ T cells, T follicular helper subsets, naïve, transitional, marginal zone (MZ), germinal center (GC), IgM memory, and class-switched memory (CSM) B cells, and T follicular regulatory, regulatory B cells, CD4 Treg, and CD8 Treg were analyzed. Serum IgG4 was elevated at 448 mg/dL. Ampula biopsy showed lamina propria fibrosis and increased IgG4-positive plasma cells. Skin punch biopsy showed lymphoplasmacytic infiltrates with a 67% ratio of IgG4+:IgG+ plasma cells. CD4+T_N_ and CD4+T_CM_ decreased, whereas CD4+T_EM_ increased. Naïve B cells increased; transitional, MZ, CSM, GC B cells, and plasmablasts decreased compared to control. CD4 Treg increased, whereas CD8 Treg and Breg decreased. In conclusion, IgG-RD may present with combined biliary tract and generalized dermatological manifestations. Changes in regulatory lymphocytes suggest their role in the pathogenesis of IgG4-RD.

## 1. Introduction

IgG4-related disorder (IgG4-RD) is a chronic fibroinflammatory disorder, characterized by elevated serum levels of IgG4 and predominant infiltration of IgG4 plasma cells, as well as the fibrosis of involved organs/tissues. The revised criteria for the diagnosis of IgG4-RD [1] include (A) clinical and radiological features of one or more organs showing diffuse or localized swelling or a mass or nodule characteristic of IgG4-RD, (B) serum IgG4 levels greater than 135 mg/dL, and (C) two of the following three pathological findings: (i) dense lymphocyte and plasma cell infiltration with fibrosis, (ii) ratio of IgG4-positive plasma cells/IgG-positive cells greater than 40% and the number of IgG4-positive plasma cells greater than 10 per high, powered field, and (iii) typical tissue fibrosis, particularly storiform fibrosis or obliterative phlebitis. The etiology of IgG4-RD disease remains unclear. Genetic, microbial, and immune factors have been proposed to play a role in the pathogenesis of IgG4-RD [2,3,4].

Hepatobiliary manifestations include IgG4-associated cholangitis, IgG4-related sclerosing cholangitis (IgG4-SC), IgG4-related hepatopathy, IgG4-related autoimmune hepatitis, and inflammatory pseudo tumors of the liver and biliary tract [5,6,7].

Skin manifestations include erythematous and itchy plaques or subcutaneous nodules on the skin in preauricule, postauricule, cheek, temporal, buttock, forearm, waist, mandible, and neck [8,9,10]. The generalized skin rashes or combined dermatological and biliary manifestations associated with IgG4-RD have not been reported.

There are limited studies on lymphocyte subsets in patients with IgG4-RD [11,12,13,14,15]. However, a comprehensive analysis of various subsets of lymphocytes has not been published. In this study, we present a unique case of IgG4-RD, diagnosed based on current criteria [1], with combined generalized skin rash and biliary tract obstruction (cholangitis). Furthermore, we report perhaps the most comprehensive analysis of various subsets of lymphocytes in IgG4-RD. They include naïve and central and effector memory subsets of CD4+ and CD8+ T cells, subsets of circulating T follicular helper cells (cT_FH_, T_FH_1, T_FH_2, T_FH_17, T_FH_1/T_FH_17), and naïve, transitional, marginal zone (MZ); germinal center (GC); IgM memory (MM); class switched memory (CSM) B cells; and plasmablasts (PB). In addition, we analyzed members of the regulatory lymphocyte club, including CD4 Treg, CD8 Treg, Breg, and T follicular regulatory (T_FR_) cells.

## 2. Materials and Methods

Peripheral blood was obtained from the patient and an age- and gender-matched healthy control.

**Figure 1:**
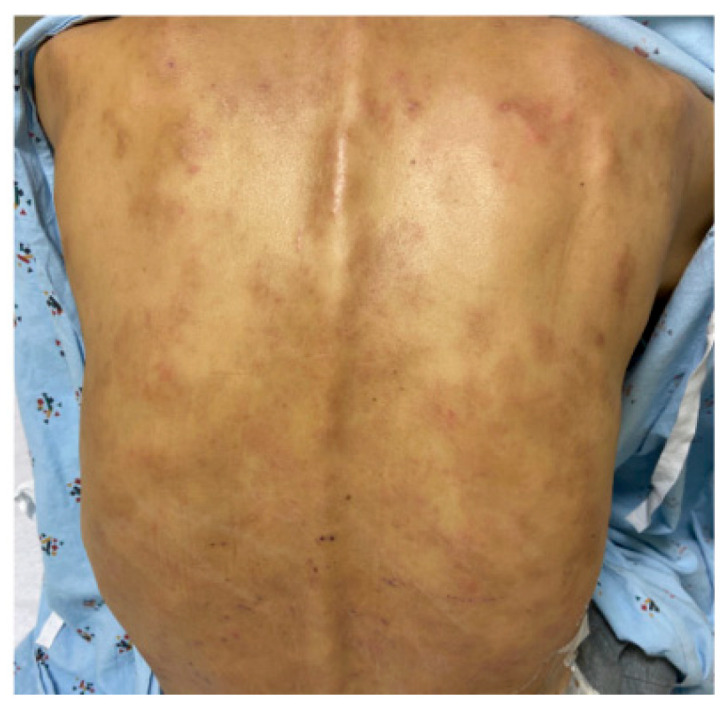
Generalized skin eruption. Pigmented generalized macular eruption on the back.

The patient is a 55-year-old Korean male who presented with fever and right upper quadrant pain, and three months later, painful jaundice. He was admitted for the fourth time with suspicion for cholangitis and was found to have a biliary obstruction. The CT abdomen and pelvis showed continuous biliary stent with stable dilated intrahepatic duct and edematous pancreas. Percutaneous cholecystostomy was carried out to alleviate gallbladder distension. Pneumobilia and intrahepatic biliary duct dilatation was observed. During stent exchange, common bile duct stricture and upstream mild common bile duct dilatation on cholangiogram were observed. The patient also reported a generalized pruritic eruption before having painful jaundice. The eruption was distributed on his upper body and face. He had erythematous ill-defined papules and thin plaques on face, chest, abdomen, and back. He also had extensive changes including excoriations, hyperpigmented patches/nodules, and lichenified thin plaques in the same locations (Figure 1).

The initial differential diagnoses by dermatologists included secondary to cholestatic pruritus and mild atopic dermatitis. Other differentials included allergic versus irritant contact dermatitis, eczematous drug eruption, and cutaneous pseudolymphoma.

His skin and ampulla were biopsied and subjected to histology and immunostaining. The patient was treated with prednisone with significant clinical improvement and normalization of serum IgG4 (from 448 mg/dL to 50 mg/dL). His laboratory investigations that were performed following treatment with steroids are shown in Table 1 and show low IgG2 and IgG3, as well as increased B cells.

### 2.1. Antibodies and Reagents

The following anti-human monoclonal antibodies and isotype controls were used to define various subsets of B cells, including anti-IgM APC, CD27 FITC, CD38 FITC, anti-IgD PE, CD21 PE, and CD27 APC purchased from BD Biosciences (San Jose, CA, USA); CD19 PerCP, CD38 APC, and CD24 FITC were purchased from BioLegend (San Diego, CA, USA). Subsets of CD4+ and CD8+ T cells were identified using the following antibodies and isotype controls, which were purchased from BD Biosciences (San Jose, CA, USA) and included CD4 PerCP, CD8 PerCP, CD45RA APC, CCR7 FITC, CD183 PE, CD25 FITC, CD127 AL647, FoxP3 PE, CD278 (ICOS) AL647, CD183 BV421, CXCR5 and PD1 APC. CD8 BV421 and CD45RA BV510 were obtained from BioLegend (San Diego, CA, USA). Subsets of circulating T Follicular helper (cT_FH_) cells: The following antibodies were used to identify different T_FH_ subsets: anti-CD4- PerCP, anti-CD45RA-BV510, anti-CXCR5-Alexa Fluor 488, anti-CCR6- PE, anti-CXCR3-BV421, anti-PD-1-PE-CF594 (all from BD Bioscience, San Diego, CA, USA), anti-ICOS-BV786 (from Biolegend, San Diego, CA, USA).

### 2.2. Flow Cytometry

Various lymphocytes were assayed by flow cytometry using multiple monoclonal antibodies and isotype controls, as previously described [16].

Approximately 1 million peripheral blood mononuclear cells (PBMCs) were used per combination for antibody staining, and 20 μL of antibody was added to PBMCs for 30 min. PBMCs were washed and fixed by 2% paraformaldehyde.

CD4 Treg, CD8 Treg, and T_FR_ cells: After surface staining, cells were fixed and permeabilized by a Foxp3 staining buffer set (BD Bioscience) as per the manufacturer’s protocol and intracellular staining with anti-Foxp3PE monoclonal antibody. The appropriate isotype control (Mouse IgG 1, k-PE) was used to evaluate nonspecific staining.

All fluorescence minus one control (FMO) and isotype controls were stained and fixed by 2% paraformaldehyde for flow cytometry. Cells were acquired by BD FACS Celesta (Becton-Dickenson, San Jose, CA, USA) and equipped with a BVR laser. Forward and side scatters and singlets were used to gate and exclude cellular debris. Thirty thousand cells were acquired and analyzed using FLOWJO 10.10.1 software (Ashland, OR, USA).

### 2.3. Surface Markers of Subsets of CD4+ and CD8+ T Cells

Different subsets of CD4 T cells and CD8+ T cells were identified by the following markers: naïve (T_N_): CD4+/CD8+CD45RA+CCR7+, central memory (T_CM_): CD4+/CD8+CD45RA−CCR7+, effector memory (T_EM_): CD4+/CD8+CD45RA−CCR7−, and terminally differentiated effector memory (T_EMRA_): CD4+/CD8+CD45RA+CCR7−.

### 2.4. Surface Markers of Subsets of Follicular Helper T Cells

Various subsets of cT_FH_ were identified using the following markers: cT_FH:_ CD4+/CXCR5+CD45RA−, T_FH_1: CD4+CXCR5+CD45RA−CCR6−CXCR3+.T_FH_2: CD4+CXCR5+CD45RA−CCR6−CXCR3−, T_FH_17: CD4+CXCR5+CD45RA−CCR6+ CXCR3−, T_FH_1+T_FH_17+:CD4+/CXCR5+/CD45RA−/CCR6+/CXCR3+.

### 2.5. Surface Markers of B Cell Subsets

The following cell surface markers were used to identify various B cell subsets as follows: naïve B cells:CD19+CD27−IgD+IgM+, transitional B cells: CD19+CD38+IgM++, MZ B cells: CD19+CD27+IgD+IgM+, IgM memory B cells: CD19+CD27+IgM+, GC B cells: CD19+IgD−CD27+CD38+, class switch memory B cells: CD19+CD27+IgD−IgM−, plasmablasts: CD19+CD38++IgM−, mature B cells: CD21^high^CD19+CD38−, CD21^Low^ B cells: CD19+CD38−CD21^low^.

### 2.6. Markers of Members of Regulatory Lymphocyte Club

All four members of the club were analyzed using following markers: CD8 Treg: CD8+CD183+CCR7+CD45RA−, CD4 Treg: CD4+CD25+CD127− Foxp3+, Breg: CD19+CD24+CD38+, and T_FR_: CD4+CCR5+CD45RA−CD25^high^FoxP3+.

## 3. Results

### 3.1. IgG4+ Plasma Cells in Skin and Ampulla Biopsy

**Figure 2:**
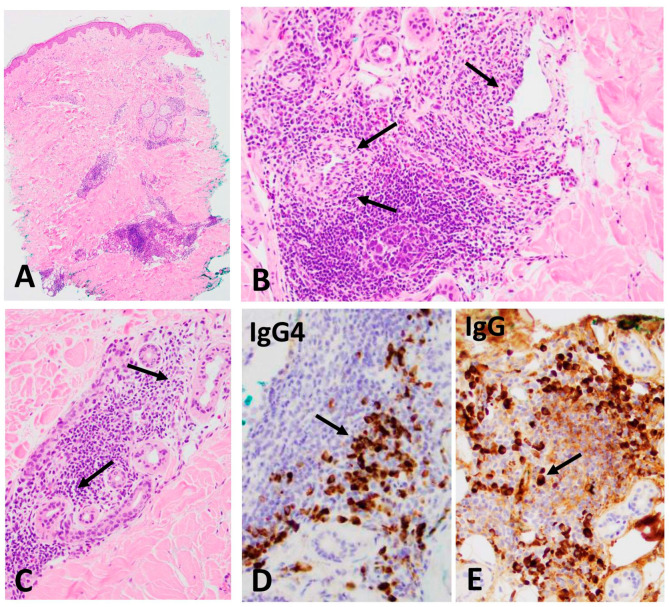
Skin Biopsy: (**A**) Scanning view showing an inflammatory infiltrate concentrated around the deep eccrine glands and small vessels (×50). (**B**) Higher power view demonstrating a dense infiltrate of lymphocytes and numerous plasma cells around the per-eccrine small vessels and eccrine glands (arrows, ×200), and periadnexal gland (**C**, arrows, ×200). Immunostains for IgG (**D**, arrow, ×400) and IgG4 (**E**, arrow, ×400) plasma cells.

Skin biopsy (Figure 2): The skin punch biopsy from an erythematous papule on the right side of trunk shows dense chronic inflammatory infiltrate concentrated around small vessels (A) and the dense infiltrate of lymphocytes and numerous plasma cells (B, arrows)in the periadnexal gland (C, arrows). Immunostains were performed for total IgG and IgG4 subclass using specific monoclonal antibodies. The ratio of IgG4-positive (D, arrow) to IgG-positive (E, arrow) plasma cells was 67%.

**Figure 3:**
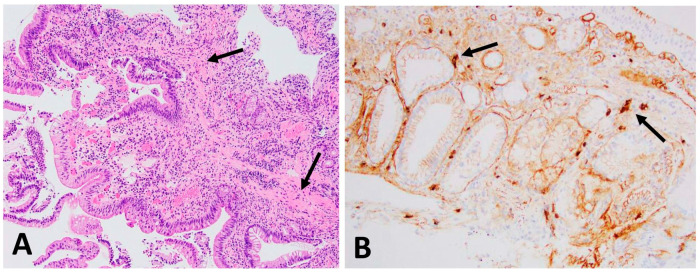
Ampulla: the low magnification of ampullary tissue shows dense infiltrates composed mostly of plasma cells. (**A**) Laminar propria showing fibrosis (**A**, arrows, ×200); immunostains show IgG4-positive plasma cells, up to 12/HPF (**B**, arrows, ×400).

*Ampulla biopsy* (Figure 3): The low magnification of ampullary tissue shows actively chronic inflammation diffusely infiltrated by lymphoplasmacytic cellularity, most of which is plasmacytes: (A) laminar propria showing fibrosis (A, arrows, ×200); immunostains demonstrated increased IgG4 positive plasma cells, up to 12/HPF (B, arrows, ×400).

### 3.2. Subsets of CD4+ and CD8+ T Cells

**Figure 4:**
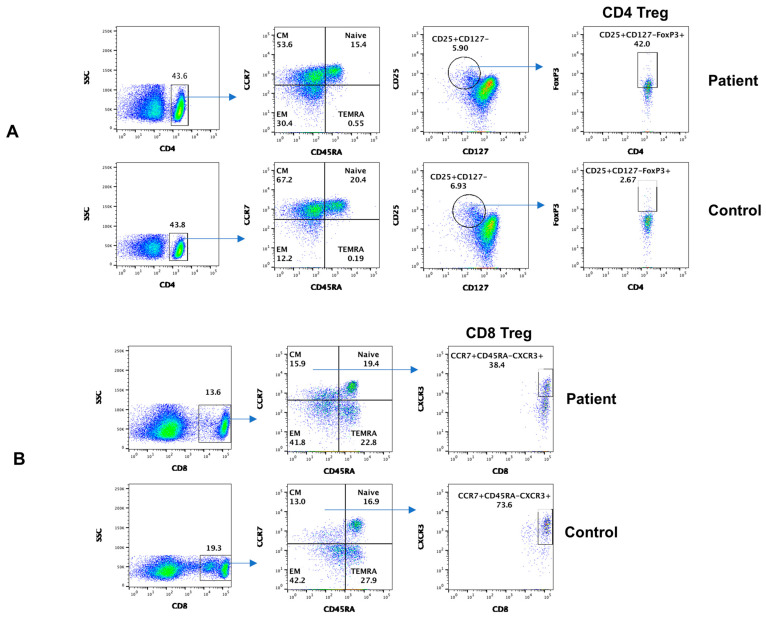
Subsets of CD4 + T cells including regulatory cells; CD4 Treg (**A**) of CD8+ T cell subsets including regulatory cells; and CD8 Treg (**B**) in the patient and control.

Subsets of CD4+ and CD8+ cells, based on the expression of chemokine receptors, homing properties, and functions, were divided into distinct subpopulations [17]. Figure 4A shows data from subsets of CD4+ T cells. CD4+T_N_ and CD4+T_CM_ decreased, whereas CD4+T_EM_ increased in the patient, as compared to control. CD4 Treg markedly increased in the patient. Data from subsets of CD8+ T cells are shown in Figure 4B. CD8+T_N_, CD8+T_CM_, CD8+T_EM_, and CD8+T_EMRA_ subsets were comparable to control; however, CD8 Treg markedly decreased.

### 3.3. Subsets of Circulating T Follicular Helper Cells (cT_FH_)

**Figure 5:**
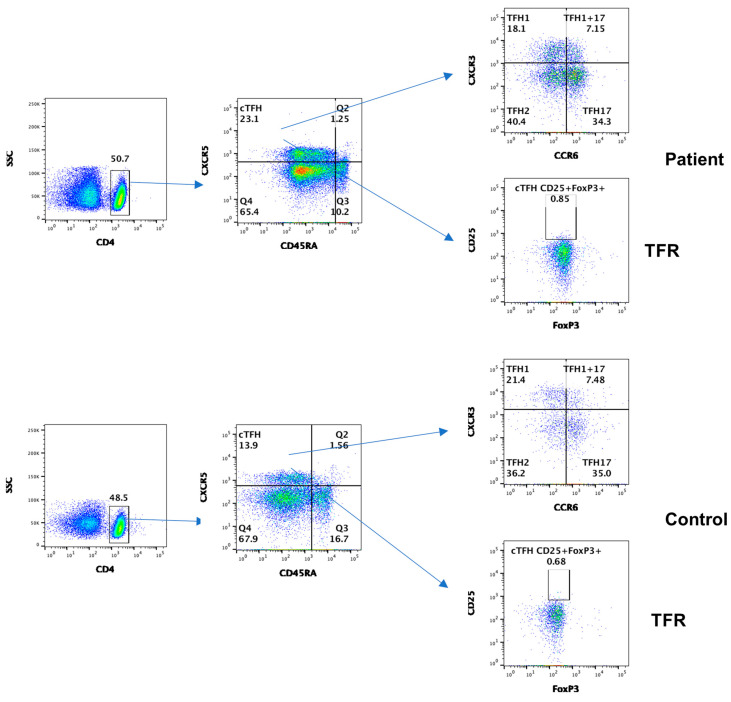
Subsets of circulating T follicular helper cells (T_FH_), including T follicular regulatory (T_FR_) cells in the patient and control.

T_FH_ play an important role in GC formation, isotype switching, and differentiation of B cells to antibody secreting plasmablasts/plasma cells [18]. Based upon the expression of CXCR3 and CCR6 on CD4 + CXCR5+ cT_FH_ cells, they are divided into different subsets with different functions and include T_FH_1, T_FH_2, T_FH_17, and T_FH_1+T_FH_17+ [19]. In addition, T follicular regulatory cells (T_FR_) regulate the function of cT_FH_ cells [20]. cT_FH_ increased whereas all other subsets in the patients, including T_FR_, were comparable to control (Figure 5).

### 3.4. Subsets of B Cells

**Figure 6:**
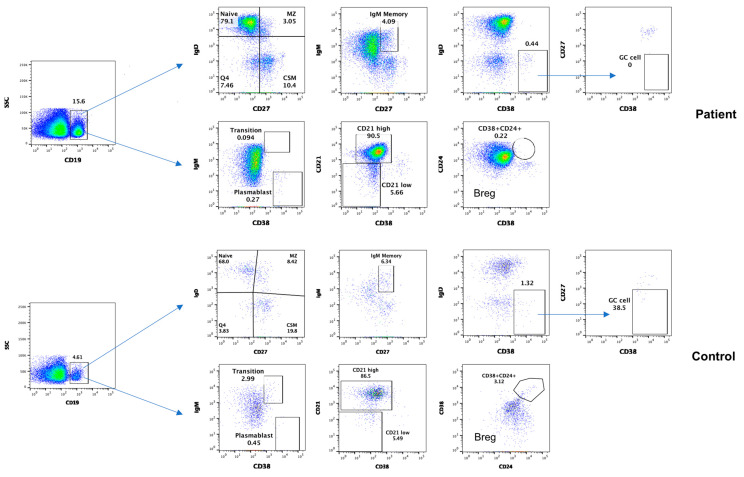
Subsets of B cells, including naïve, transitional, marginal zone (MZ), germinal center B cells (GC), IgM memory, class-switched memory (CSM), plasmablasts, CD21^low^ and regulatory B cells (Breg) in the patient and control.

Data from B cell subsets are shown in Figure 6. Naïve B cells increased, whereas other subsets of B cells, including transitional zone, MZ, CSM, and GC B cells, plasmablasts, and Breg, decreased compared to the simultaneously analyzed healthy control.

## 4. Discussion

We present the first case of IgG4-related generalized skin rash and first case of combined skin and biliary tract manifestations, and perhaps the most comprehensive analysis of lymphocyte subsets in IgG4-RD. Our patient met all diagnostic criteria for IgG4-RD including radiological findings, elevated serum IgG of more than 135 mg/dL (448 mg/dL), dense lymphocyte and plasma cell infiltration with fibrosis, more than 10 plasma cells/HPF (12/HPF), and 40% or more (67%) IgG4 plasma cells of total IgG plasma cells. Although chronic pancreatitis associated with hyperimmunoglobulinemia was described in 1961, it was not until 2001 that Hamano and colleagues [21] reported high serum IgG4 and infiltration of the pancreas with IgG4+ plasma cells. Thereafter, it became apparent that 27 multiple organs, including the salivary gland, lacrimal gland, kidneys, lungs, thyroid, pituitary gland, prostate gland, liver and biliary tract, and lymph nodes, display increased IgG4+ plasma cells and evidence of fibrosis in patients with elevated serum IgG4 [22]. In 2012, a recommendation regarding the nomenclature for IgG4-RD [23] and consensus on the pathology of disease were established [24]. The criteria were revised in 2020 [1]. IgG4-related sclerosing cholangitis is the most common extrapancreatic manifestation of IgG4-RD [25]. Sclerosing cholangitis is commonly seen in association with autoimmune pancreatitis and intrahepatic disease [26,27], and only between 1.5% [16] and 8% [4] of patients have isolated sclerosing cholangitis. In our patient, cholangitis was not associated with autoimmune pancreatitis or hepatopathy.

The involvement of skin IgG4-RD appears to be underestimated. Yamada et al. [9] reported skin involvement in 6% of IgG4-RD cases. Katerji and Smoller [10] recently reviewed skin manifestations in IgG-RD. These skin lesions have been associated with chronic sclerosing dacryoadenitis and sialadenitis or systemic IgG4-related lymphadenopathy [28,29]. Sato et al. [29] showed that 3 of 9 patients with systemic IgG4-related lymphadenopathy had skin lesions and demonstrated that one of them had cutaneous pathological findings typical of IgG4-RD. Sato et al. [8] reported a clinicopathological analysis of 10 patients with IgG4-related skin lesions disease. In all reported cases of IgG4-related skin disease, the skin lesions are localized to face, head, and neck, particularly the preauricular cheek and mandible region. Our patient is unique in that he has diffused generalized skin eruption associated with IgG4-related cholangitis. There was no enlargement of salivary or lacrimal glands or associated lymphadenopathy.

The etiology of IgG4-RD is unclear; however, genetic and immunological factors are reviewed in [5,30]. The evidence of IgG4 itself causing the pathology of IgG4-RD is lacking.

A number of both non-specific and specific autoantibodies directed against pancreatic antigens are often detected in patients with IgG4-RD; these include ANA, RF, and low levels of anti-DNA antibodies [22,30]. However, these autoantibodies are not related to the clinical features seen in IgG4-related disease. The autoantibodies against pancreatic antigens, anti- carbonic anhydrase II antibodies, anti-lactoferrin antibodies, and anti-pancreatic secretory trypsin inhibitor antibodies are also not highly specific for this disease. There is no direct evidence that autoantibodies have a role in the pathogenesis of IgG4-related disease. In our patient, autoantibodies screened including ANA, ANCA, SSA, and SSB were negative.

The role of T cells, Tregulatory cells (Treg), follicular helper (T_FH_) cells, and CD4 cytotoxic T cells (CD4+CTL) in the pathogenesis of IgG4RD has been researched [11,12,13,14,15,31,32].

T_FH_ cells interact with B cells and play a critical role in the formation of germinal center, immunoglobulin isotype switching, and affinity maturation [18]. Based on the expression of CXCR3 and CCR6, T_FH_ cells were divided into several subsets [19]. Kubo et al. [11] and Chen et al. [12] reported increased T_FH_2 in IgG4-RD. Akiyama et al. [13] reported increased T_FH_1 and T_FH_2 cells in IgG4-RD, and both were decreased following glucocorticoid treatment. In our patient, we performed a comprehensive study of cT_FH_ cells and subsets, including T_FH_1, T_FH_2, T_FH_17, T_FH_1/17, and T_FR_ cells. The circulating cT_FH_ cells were increased, whereas T_FH_1, T_FH_2, T_FH_17, and T_FH_1/17 subsets in patients were comparable to control.

Mattoo et al. [31,32] suggested a role of CD4+CTL in the pathogenesis of IgG4-RD. Using next-generation sequencing, they observed an expansion of CD4+CTL cells both in the blood and in the tissue lesions. These cells produce transforming growth factor, IFNγ (mediator of fibrosis), IL-4, and IL-5 (mediators of IgE and eosinophilia).

Increased serum IgG4 and the infiltration of tissues and organs involved with IgG4+ plasma cells suggest a role of B cells and antibodies in the pathogenesis of IgG4-related diseases. Lin et al. [14] studied peripheral B cell subsets, including plasmablasts, memory B cells, naïve B cells, and regulatory B cells in untreated patients with IgG4-related diseases. They observed that CD19+ CD24− CD38hi plasmablast/plasma cells were increased and positively correlated with serum IgG4 levels, the number of involved organs. Lighaam et al. [15] reported an increase in IgG4 memory B cells and plasmablasts in the blood in active disease. Lin et al. [14] reported that plasmablasts dramatically decreased following treatment with corticosteroids. In our patient, all subsets of B cells including GC and plasmablasts decreased, which may have been due in part to corticosteroids treatment that he received prior to investigation.

Regulatory lymphocytes play an important role in immune homeostasis and regulation of autoimmunity. The members of the regulatory lymphocyte club include CD4 Treg, CD8 Treg, Breg, and T_FR_ cells. Sakaguchi and his group characterized CD4+ Treg using the expressions of CD25 and FoxP3 [33]. There is no study of all regulatory lymphocytes in IgG4-RD. The number of CD4 Treg cells in biliary tissue correlates with tissue IgG+ plasma cells in IgG4-related sclerosing cholangitis, and circulating CD4 Treg cells correlate with serum IgG4 in autoimmune pancreatitis [34]. In our patient, we also observed increased CD4 Treg.

CD8 Treg cells have never been reported in IgG4-RD. CD8 Treg cells selectively inhibit plasmablasts and inhibit B cell proliferation and immunoglobulin production [35,36]. Our patient displayed decreased CD8 Treg, which could play a role in increased plasmablasts and elevated serum IgG4 in IgG4-RD.

T_FR_ cells regulate antibody production by regulating the function of T_FH_ [20]. We did not find any difference in T_FR_ cells in our patient. However, as compared to control, Fumie and colleagues [37] reported increased T_FR_ cells in the blood and submandibular glands from patients with IgG4-RD, and percentages of T_FR_ correlated with serum IgG and the number of involved organs in IgG4-RD. This appears to be a paradox because T_FR_ cells regulate the functions of T_FH_ cells and antibody production. Thus, increased T_FR_ cells would have been associated with decreased autoantibody production.

Breg play an important role in immune homeostasis and in the regulation of autoimmunity and autoimmune diseases [38] and regulate the generation of peripheral CD4+ Treg cells. Therefore, decreased Breg may contribute to increased CD4+ Treg in our patient. Our observations are similar to those of Lin and associates [23], who also reported decreased Breg in patients with IgG4-RD.

## 5. Conclusions

IgG4-RD may present with generalized skin eruption associated with cholangitis and perhaps involvement with other organs. IgG4-RD is associated with alterations in phenotypically characterized subsets of CD4+, CD8+, T_FH_, B cells, and regulatory lymphocytes. Our patient was found to have to have regulatory lymphocytes that could potentially play a role in the pathogenesis of IgG4-RD manifestations. However, a larger number of patients need to be studied to draw a definitive conclusion regarding a role of T cell subsets and regulatory lymphocytes in the pathogenesis of IgG4RD.

## Figures and Tables

**Table 1:** Laboratory features of IgG4-RD patient.

Test	Patient	Reference Ranges
Absolute lymphocyte counts	3774	900–3300
HepBs IgG antibodies	Positive	Negative
HepC antibodies	Negative	Negative
Autoantibodies:		
ANA	Negative	Negative < 1:40
ANCA	Negative	Negative
SSA	Negative	Negative
SSB	Negative	Negative
Serum Immunoglobulins (mg/dL)		
IgA	106	68–378
IgM	45	37–318
IgG	728	610–1616
IgG1	247	239–1083
IgG2	34	148–548
IgG3	16	27–134
IgG4	448 (50 #)	5–125
Specific antibodies		
Strep. Pneumoniae (Protected titers > 1.3 µg/mL)		
	20/23	17–21/23
Isohemagglutinins	1:8 *	1:16–1:64
Lymphocyte subsets% (numbers)		
CD3+ T cells	61 (1159)	62–84 (619–1847)
CD4+ T cells	47 (895)	31–61 (338–1194)
CD8+ T cells	14 (66)	10–38 (85–729)
CD19+ B cells	36 (684) **	5–26 (51–473)
CD3−CD56+CD16+ NK	2 (38)	1–17 (12–349)
Interleukin-6	normal	

# following steroid treatment., * decreased; ** increased.

## Data Availability

All data are included in the manuscript. For further corresponding information, the authors may be contacted.
